# pH-Responsive Redox Nanoparticles Protect SH-SY5Y Cells at Lowered pH in a Cellular Model of Parkinson’s Disease

**DOI:** 10.3390/molecules26030543

**Published:** 2021-01-21

**Authors:** Monika Pichla, Grzegorz Bartosz, Ireneusz Stefaniuk, Izabela Sadowska-Bartosz

**Affiliations:** 1 Laboratory of Analytical Biochemistry, Institute of Food Technology and Nutrition, College of Natural Sciences, Rzeszow University, 4 Zelwerowicza Street, 35-601 Rzeszow, Poland; monika.pichla@outlook.com; 2 Department of Bioenergetics, Food Analysis and Microbiology, Institute of Food Technology and Nutrition, College of Natural Sciences, Rzeszow University, 4 Zelwerowicza Street, 35-601 Rzeszow, Poland; gbartosz@ur.edu.pl; 3 Teaching and Research Center of Microelectronics and Nanotechnology, College of Natural Sciences, University of Rzeszow, 35-959 Rzeszow, Poland; istef@univ.rzeszow.pl

**Keywords:** pH-responsive redox nanoparticles, human neuroblastoma SH-SY5Y cells, Parkinson’s disease, 6-hydroxydopamine

## Abstract

The damage to SH-SY5Y cells by 6-hydroxydopamine (6-OHDA) is an established cellular model of Parkinson’s disease (PD). Redox nanoparticles are a promising tool for therapy, including neurodegenerative diseases. As pH of the brain tissue at sites affected by PD is lowered down to 6.5, we studied the effect of pH-responsive redox nanoparticles (poly(ethylene glycol)-*b*-poly[4-(2,2,6,6-tetramethylpiperidine-1-oxyl)aminomethylstyrene]), which change their structure in a pH-dependent manner and become active below pH 7 (NRNPs ^pH^), on the viability of SH-SY5Y cells treated with 6-OHDA at pH 6.5 and 7.4. Pretreatment of the cells with NRNPs ^pH^ (15–75 μM) prior to the 6-OHDA treatment increased their survival in a concentration-dependent manner at pH 6.5, but not at pH 7.4. Among several parameters studied (ATP and GSH content, the level of reactive oxygen species, mitochondrial potential, mitochondrial mass), only the mitochondrial mass was dose-dependently protected by NRNPs ^pH^ at pH 6.5, but not at pH 7.4. These results indicate that the action of NRNPs ^pH^ on mitochondria underlies their protective effect in this cellular model of PD. These results may have potential importance for future applications of NRNPs ^pH^ in preclinical and perhaps clinical studies.

## 1. Introduction

It is astonishing that, despite the extraordinary progress of medicine, the treatment of neurodegenerative diseases has not progressed for over half a century, and still is purely symptomatic [1]. Where does the blame lie? Therapeutics do not have an easy road when it comes to reaching a target site. There are numerous biological obstacles that hinder the effective delivery of drugs, e.g., non-specificity, inadequate accumulation, opsonization, and sequestration via mononuclear phagocyte system, drug efflux pumps, evading lysosomal and endosomal compartments and general cellular internalization [2]. Thankfully, nanomedicine can offer help in overcoming these blockades and delivering cargo to particular intracellular regions. Redox-active nanoparticles of various structure scavenging reactive oxygen species (ROS) have been proposed to combat oxidative stress [3,4]. A promising approach to face these obstructions is a use of nanoparticles designed to undergo modifications in material properties as a result of exposure to different internal or external stimuli such as: pH, redox state [5,6,7], enzymatic activity [8], temperature, magnetic or electric field, ultrasounds or light [9].

The pH-sensitive have started to gain research interest, because they can be exploited at three levels, namely organ, tissue, and cellular level, in order to increase and improve drug uptake by the gastrointestinal tract, take advantage of tumor microenvironment to advance drug specificity and lastly, profit from the “proton sponge” effect to internalize drugs, respectively [10]. Nanoparticles can be engineered to change their size, shape of surface chemistry in response to the stimuli, to disassemble or release the load. This can be achieved by various strategies. One of them is the exploitation of charge shifting compounds, next, the use of acid labile linkages or crosslinkers [11]. The importance and possibilities of the use of nanoparticles against neurodegeneration have been reviewed elsewhere [12,13].

Neurodegenerative diseases are complex and the exact etiopathogenesis is still unknown [14], which does not help to create fully reliable disease models. Nevertheless, mitochondrial dysfunction is an inseparable part of neurodegenerative diseases. It causes a drop in pH by the leakage of protons into the cytosol [15], hence a decreased pH of brain tissue might be exploited to target the treatment, similarly as the tumor microenvironment. Data provided by Genoud et al. indicate that average pH of human brain tissue affected by Parkinson’s disease (PD) is as low as ~6.5 [16].

The pH-sensitive antioxidant-loaded nanoparticles (poly(ethylene gly-col)-*b*-poly[4-(2,2,6,6-tetramethylpiperidine-1-oxyl)aminomethylstyrene] nanoparticles) possessing nitroxide radicals as a side chain of a polymer segment, called further NRNPs ^pH^, were designed and developed for selective action at lower pH. These nanoparticles in aqueous solution have a form of self-assembling polymeric micelles, which disintegrate in the acidic environment, by virtue of the protonation of amino groups present in their core [17,18]. NRNPs ^pH^ were employed to effectively scavenge overproduced ROS in inflamed tissues and cancerous regions, to improve anticancer efficacy of doxorubicin in the colitis-associated mice colon cancer, and to decrease cardiac levels of ROS in doxorubicin-treated mice [19]. They showed also a protective effect in renal ischemia-reperfusion in experimental animals [18]. The transgenic Tg2576 Alzheimer’s disease (AD) mice treated with NRNPs ^pH^ had significantly attenuated cognitive deficits of both spatial and non-spatial memories, reduced oxidative stress, and decreased lipid peroxides as well as DNA oxidation [20]. The effect of NRNPs ^pH^ in PD has not been studied so far. Therefore, we decided to check their action in the cellular model of this disease.

## 2. Results

### 2.1. Penetration of pH-Sensitive NRNPs into SH-SY5Y Cells

**Figure 1:**
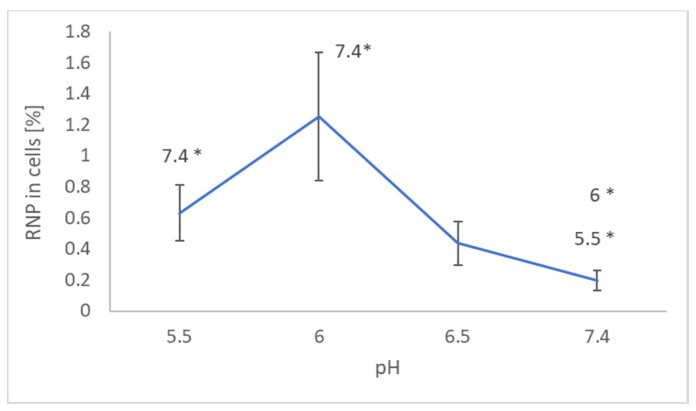
Effect of pH on the uptake of NRNPs ^pH^ by SH-SY5Y cells. * *p* < 0.05 with respect to the pH indicated, Student *t*-test; *n* = 3.

Measurements of EPR signal of NRNPs ^pH^ in cells and in supernatants showed that the uptake of nanoparticles was maximal at pH 6.0 (Figure 1).

**Figure 2:**
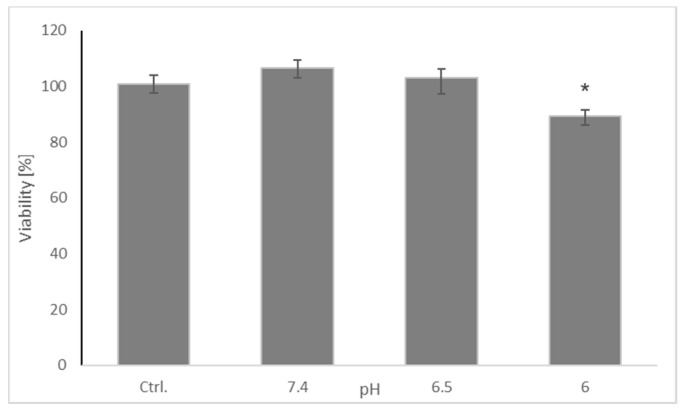
Effect of pH on the viability of SH-SY5Y cells. Cell viability was estimated with Presto Blue. The whiskers are lower (25%) and upper (75%) quartile ranges. * *p* ≤ 0.05, Kruskal–Wallis test vs. control (Ctrl); *n* = 9.

Viability of the cells did not show significant differences between pH 7.4 and 6.5 but was decreased at pH 6.0 (Figure 2). For this reason, and, taking into account that the pH in the affected regions of the brain may be lowered to about 6.5, the pH of 6.5 was compared with pH 7.4 in subsequent experiments.

Monitoring time course of the uptake revealed no discernible time dependence of the uptake for incubation times longer than 3 h, indicating rapid uptake by the cells. Therefore, the time of 2 h was chosen for cell pretreatment with the NRNPs ^pH^ in further experiments.

### 2.2. Cell Viability

**Figure 3:**
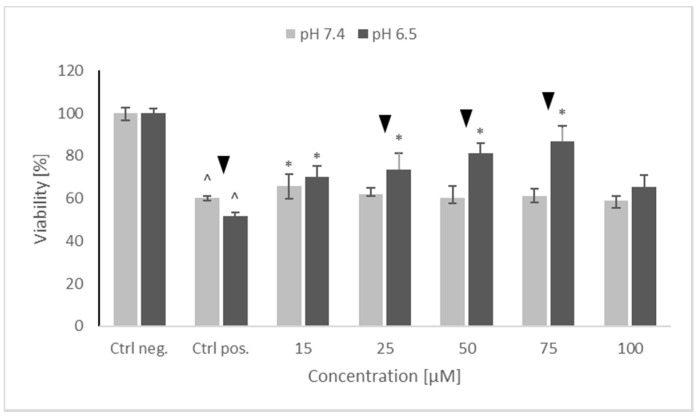
The protective properties of NRNPs ^pH^ against 65 μM 6-hydroxydopamine-induced cytotoxicity for SH-SY5Y cells at pH 7.4 and pH 6.5. Cell viability was estimated with MTT. The whiskers are lower (25%) and upper (75%) quartile ranges. ^ *p* ≤ 0.05, Kruskal–Wallis test vs. the negative control (Ctrl neg.; cells not treated with 6-OHDA), ▼ *p* ≤ 0.05, Mann–Whitney U test; differences between the different pH conditions (pH 7.4 vs. pH 6.5), * *p* ≤ 0.05, Kruskal–Wallis test vs. positive control (Ctrl pos.; cells treated with 6-OHDA only, without any pretreatment); *n* = 9.

As aforementioned in our previous studies, oxidopamine (also known as 6-hydroxydopamine; 6-OHDA) showed a dose-dependent cytotoxicity against SH-SY5Y cells [21]. 6-Hydroxydopamine (65 µM) was somewhat more cytotoxic at pH 6.5 than at pH 7.4. NRNPs ^pH^ did not exhibit a statistically significant self-cytotoxicity up to ca. 75 μM at both pH conditions (not shown). However, they showed a concentration-dependent protection against 6-OHDA-induced cytotoxicity at both pH values studied and were more efficient at pH 6.5. At pH 7.4, NRNP ^pH^ restored the viability of 6-OHDA treated cells up to about 65% of the control value while at pH 6.5 up to ca. 85% of the control value (Figure 3).

### 2.3. Intracellular ATP Level

**Figure 4:**
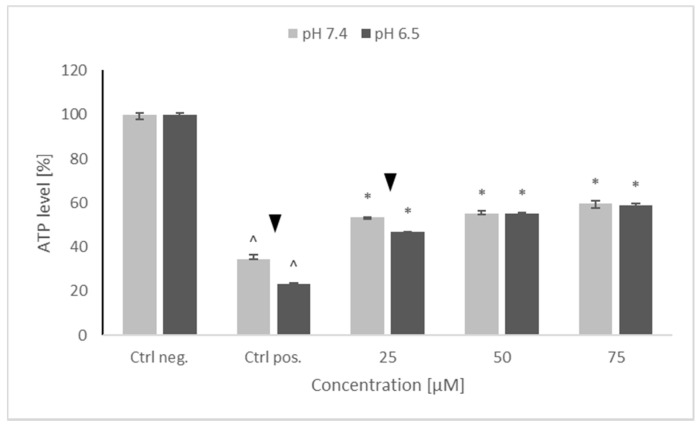
ATP levels after pretreatment of SH-SY5Y cells with NRNPs ^pH^ and exposure to 6-hydroxydopamine. The whiskers are lower (25%) and upper (75%) quartile ranges. ^ *p* ≤ 0.05, Kruskal–Wallis test vs. the negative control (Ctrl neg.; cells not treated with 6-OHDA),▼ *p* ≤ 0.05, Mann–Whitney U test; differences between the different pH conditions (pH 7.4 vs. pH 6.5), * *p* ≤ 0.05, Kruskal–Wallis test vs. positive control (Ctrl pos.; cells treated with 6-OHDA only, without any pretreatment); *n* = 9.

Treatment with 6-OHDA induced a significant drop in the ATP level of the SH-SY5Y cells, more pronounced at pH 6.5 than at pH 7.4. Preincubation with NRNPs ^pH^ partly prevented this drop and, starting from the concentration of 50 μM, the ATP level was preserved at the same level at both pH values. However, maximal ATP level in the cells pretreated with NRNPs ^pH^ and challenged with 6-OHDA did not exceed 60% of the level of the negative control (cells not treated with 6-OHDA) (Figure 4).

### 2.4. Content of Reduced Glutathione

**Figure 5:**
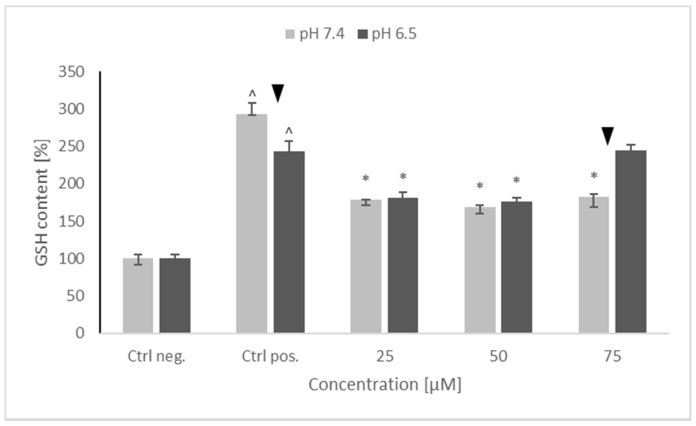
Glutathione (GSH) content of SH-SY5Y cells after pretreatment with NRNPs ^pH^ and exposure to 6-hydroxydopamine. The whiskers are lower (25%) and upper (75%) quartile ranges. ^ *p* ≤ 0.05, Kruskal–Wallis test vs. the negative control (Ctrl neg.; cells not treated with 6-OHDA), ▼ *p* ≤ 0.05, Mann–Whitney U test; differences between the different pH conditions (pH 7.4 vs. pH 6.5), * *p* ≤ 0.05, Kruskal–Wallis test vs. positive control (Ctrl pos.; cells treated with 6-OHDA only, without any pretreatment); *n* = 9.

A substantial increase in GSH content was seen in positive control (cells treated with 6-OHDA only, without any pretreatment). Pretreatment with NRNPs ^pH^ decreased the GSH content nearly by half in the 25–75 μM concentration range at pH 7.4, and at 25 μM and 50 μM concentrations at pH 6.5. However, at pH 6.5, NRNPs ^pH^ at a 75 μM concentration did not decrease the GSH content. Furthermore, no difference in the efficacy of nanoparticles was seen between the two pH conditions, except for the 75 μM NRNPs ^pH^ (Figure 5).

### 2.5. ROS Levels Using the Dihydroethidine (DHE) Fluorescent Probe

**Figure 6:**
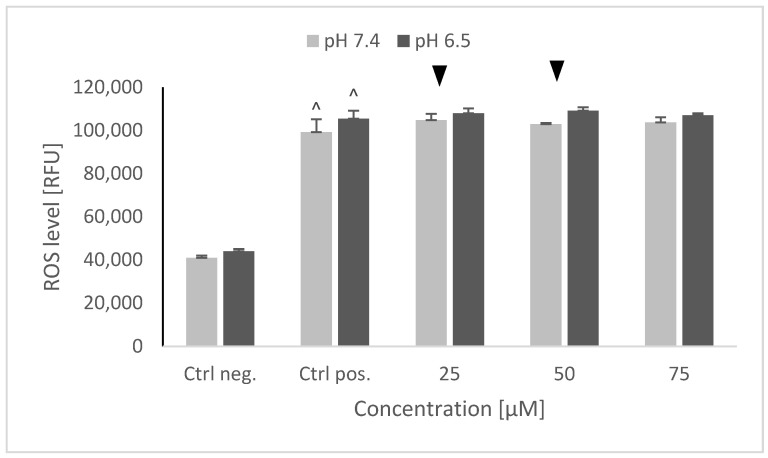
ROS level in SH-SY5Y cells determined using dihydroethidine (DHE) after pretreatment with NRNPs ^pH^ and exposure to 6-hydroxydopamine. The whiskers are standard deviation. ^ *p* ≤ 0.05, Student *t*-test vs. the negative control (Ctrl neg.; cells not treated with 6-OHDA), ▼ *p* ≤ 0.05, paired Student *t*-test, differences between the different pH conditions (pH 7.4 vs. pH 6.5), (Ctrl pos.; cells treated with 6-OHDA only, without any pretreatment); *n* = 3.

6-hydroxydopamine caused a significant, up to a 2.5× increase of the ROS level, which was not affected by the cell pretreatment with and NRNPs ^pH^ at both pH values (Figure 6).

### 2.6. Mitochondrial Membrane Potential (ΔΨ_m_)

**Figure 7:**
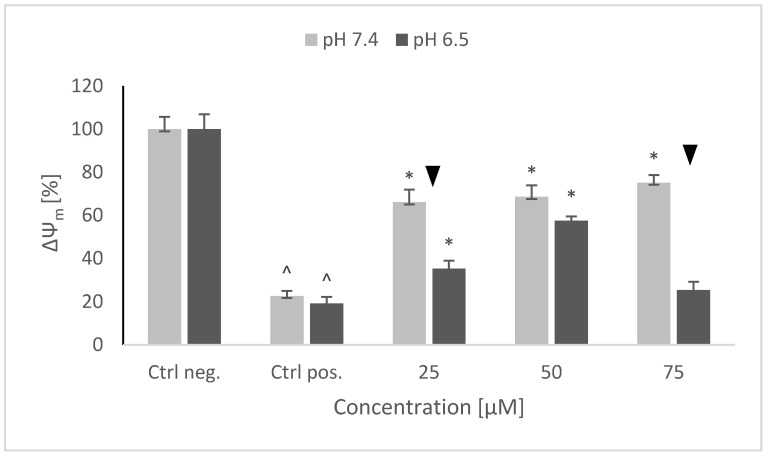
Changes in the mitochondrial potential of SH-SY5Y cells after pretreatment with NRNPs ^pH^ and exposure to 6-hydroxydopamine. The whiskers are standard deviation. ^ *p* ≤ 0.05, Student *t*-test vs. the negative control (Ctrl neg.; cells not treated with 6-OHDA), ▼ *p* ≤ 0.05, paired sample Student *t*-test, differences between the different pH conditions (pH 7.4 vs. pH 6.5), * *p* ≤ 0.05, Student *t*-test vs. the positive control (Ctrl pos.; cells treated with 6-OHDA only, without any pretreatment); *n* = 3.

A strong decrease of the mitochondrial membrane potential was observed after 6-OHDA treatment, similar at both pH values, the ratio of red to green fluorescence being lowered by about 80%. NRNPs ^pH^ pretreatment prevented this decrease in a concentration-dependent manner in the whole range of studied concentrations at pH 7.4, and at 25 and 50 μM at pH 6.5. The protective effect was lowered at pH 6.5 (Figure 7).

### 2.7. Mitochondrial Mass

**Figure 8:**
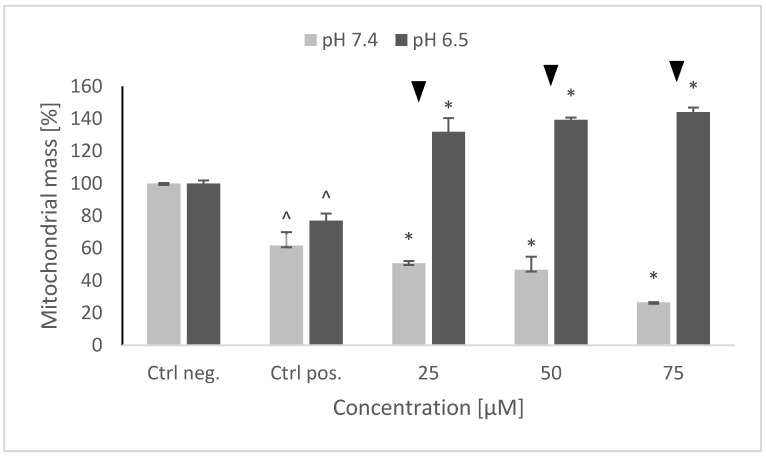
Changes in the mitochondrial mass after pretreatment of SH-SY5Y cells with NRNPs ^pH^ and exposure to 6-hydroxydopamine. The whiskers are standard deviation. ^ *p* ≤ 0.05, Student *t*-test vs. the negative control (Ctrl neg.; cells not treated with 6-OHDA), ▼ *p* ≤ 0.05, paired Student *t*-test, differences between (pH 7.4 vs. pH 6.5), * *p* ≤ 0.05, Student *t*-test with respect to the positive control (Ctrl pos.; cells treated with 6-OHDA only, without any pretreatment); *n* = 3.

6-hydroxydopamine induced a drop in the mitochondrial mass in the SH-SY5Y cells. Pretreatment with NRNPs ^pH^ had a clearly distinct effect on the mitochondrial mass depending on pH. While at pH 7.4 NRNPs ^pH^ caused a further concentration-dependent decrease of the mitochondrial mass, they increased concentration-dependently the mitochondrial mass at pH 6.5 (Figure 8).

### 2.8. Prevention of Apoptosis and Necrosis

**Figure 9:**
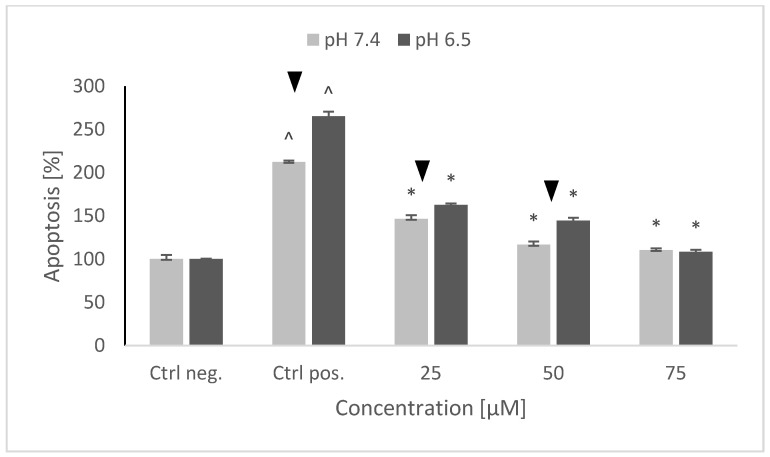
The level of apoptosis of SH-SY5Y cells after pretreatment with NRNPs pH and exposure to 6-hydroxydopamine. The whiskers are standard deviation. ^ *p* ≤ 0.05, Student *t*-test vs. the negative control (Ctrl neg.; cells not treated with 6-OHDA), ▼ *p* ≤ 0.05, paired Student *t*-test, differences between different pH conditions (pH 7.4 vs. pH 6.5), * *p* ≤ 0.05, Student *t*-test vs. the positive control (Ctrl pos.; cells treated with 6-OHDA only, without any pretreatment); *n* = 3.

Treatment of SH-SY5Y cells with 6-OHDA strongly augmented both necrosis and apoptosis. It is not possible to determine the contribution of both cell death types, since the used test allows only for a relative estimation of apoptosis and necrosis levels. Though the increase in the rate of necrosis was higher than that in the rate of apoptosis, it is hard to say what was the absolute level of apoptosis and necrosis in the control preparations (the level of necrosis could be much lower with respect to apoptosis). 6-OHDA induced a higher increase in the rate of apoptosis at pH 6.5 with respect to pH 7.4 and a higher increase in necrosis at pH 7.4 with respect to pH 6.5. Pretreatment with NRNPs ^pH^ decreased the rate of apoptosis in a concentration-dependent manner. Lower concentrations—25 and 50 μM NRNPs ^pH^—were more effective at pH 7.4 than at pH 6.5, but the efficacy of 75 μM nanoparticles was the same at both pH values (Figure 9).

**Figure 10:**
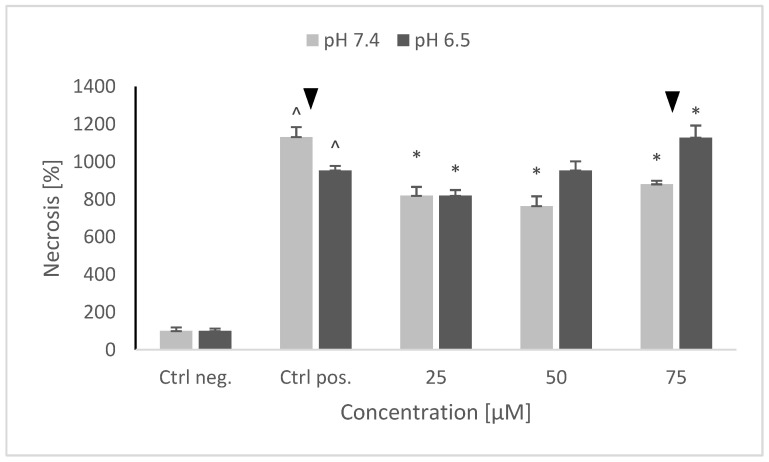
The level of necrosis after pretreatment of SH-SY5Y cells with NRNPs ^pH^ and exposure to 6-hydroxydopamine. The whiskers are standard deviation. ^ *p* ≤ 0.05, Student *t*-test vs. the negative control (Ctrl neg.; cells not treated with 6-OHDA), ▼ *p* ≤ 0.05, paired Student *t*-test, differences between different pH conditions, * *p* ≤ 0.05, Student *t*-test vs. the positive control (Ctrl pos.; cells treated with 6-OHDA only, without any pretreatment); *n* = 3.

NRNPs ^pH^ also decreased the rate of necrosis at pH 7.4, but this effect was not concentration-dependent. At pH 6.5, NRNPs ^pH^ were the most effective at the lowest concentration applied (25 μM); the protective effect was lower at higher concentrations (Figure 10).

## 3. Discussion

A spectrum of various factors underlies the physiopathology of PD, i.e., genetic factors, impaired signal transduction involved in maintaining mitochondrial homeostasis, impairment of regulation of protein degradation (proteasomal degradation and autophagy) and controlling pathways that help to maintain redox homeostasis [22,23]. Due to the complexity of mechanisms that constitute the basis of all PD characteristics, the understanding of the molecular mechanisms of this disease is far from complete, and its treatment is purely symptomatic and has not been substantially modified for over six decades [1]. Nevertheless, there is a consensus that mitochondrial dysfunction and elevated ROS levels are an integral part of PD pathophysiology, leading to the damage of dopaminergic neurons, thus antioxidants may serve as a useful approach to alleviate PD symptoms [24,25]. Several cellular models to mimic PD have been proposed, based on the effects of compounds inducing oxidative stress to neuronal cells in vivo; one of the most popular among them employs the catecholaminergic neuroblastoma cell line SH-SY5Y treated with the catecholaminergic neurotoxin 6-OHDA [26,27]. Although the 6-OHDA model does not cover all PD symptoms, it does reproduce the main cellular processes involved in PD. The model has been widely used at physiological pH (7.4). However, since the brain tissue affected by PD has pH lowered to about 6.5 [16], it seemed reasonable to check the protection of SH-SY5Y cells against the effects of 6-OHDA at the lowered pH. NRNPs ^pH^ seemed to be an appropriate candidate for such protective agent. They are able to cross the blood–brain barrier [20] and reach the brain regions affected by PD. They change their structure in response to the lowering of pH below 7, exposing 4-*N*-linked 2,2,6,6-tetramethylpiperidine-*N*-oxyl redox active residues contained within the hydrophobic shell at higher pH [21,28].

Penetration of NRNPs ^pH^ into the SH-SY5Y cells strongly depended on pH, showing optimum at pH 6. More NRNPs ^pH^ penetrated the cells at pH 6.5 than at pH 7.4 (Figure 1). Apparently, unmasking the positive charge of the nanoparticles facilitates their adsorption on the negatively charged cell surface and further penetration of the plasma membrane.

Preincubation of SH-SY5Y cells with NRNPs ^pH^ (15–75 μM) before exposure to 6-OHDA protected the cells at pH 6.5, whereas the protection at pH 7.4 was negligible (Figure 3). The protection was mainly associated with an inhibition of apoptosis, which occurred at both pH values; however, its rate was higher at pH 6.5 (Figure 9). In order to get an insight into the mechanism of cell protection at pH 6.5, we compared the effect of NRNPs ^pH^ on selected biochemical parameters of the cells. Even though nanoparticles protected the SH-SY5Y cells against the 6-OHDA-induced drop of ATP levels, this effect did not differ significantly at both pH values. The only exception was the lower protective effect at 25 μM concentration of NRNPs ^pH^ at pH 6.5 (Figure 4).

Interestingly, 6-OHDA treatment induced an increase rather than a decrease of cellular GSH (Figure 5). This effect has been observed by us previously [21] and is apparently due to the an overcompensative reaction to the initial GSH depletion by 6-OHDA as demonstrated by others [29]. The adaptive increase in the GSH level was prevented to the same extent by NRNPs ^pH^ at pH 7.4 and pH 6.5, except for a lower effect at pH 6.5, at a 75 µM concentration (Figure 5). The increase in the DHE-detectable ROS level (mainly superoxide) was not affected by preincubation with NRNPs ^pH^ (Figure 6). Cells were more protected from the mitochondrial depolarization induced by 6-OHDA at pH 7.4 than at pH 6.5 (Figure 7). Only the decrease of mitochondrial mass was prevented in a concentration-dependent manner by NRNPs ^pH^ at pH 6.5, and it significantly lessened at pH 7.4 (Figure 8).

Positively charged NRNPs ^pH^ can be expected to accumulate in the most negatively charged site of the cell, i.e., inside mitochondria. Therefore, mitochondrial effects of these particles may be anticipated. Such a localization of NRNPs ^pH^ may limit or prevent their action outside mitochondria such as scavenging of ROS outside mitochondria.

Mitochondria play several important roles in the cell. Apart from being the main ATP producer and the main source of ROS, they are involved, i.a., in the control of cell cycle, apoptosis, heme, and steroid synthesis [30,31]. All of these processes can be affected by 6-OHDA and, depending on the damage extent, may be critical for cell survival. Although the exact mechanism of SH-SY5Y cell protection against the 6-OHDA cytotoxicity cannot be inferred from this study, the obtained results indicate that stimulation of mitochondrial biogenesis by NRNPs ^pH^ can evidently prevent the outcomes of the cell survival-limiting damage. Animal experiments are foreseen to check the efficacy of NRNPs ^pH^ in an in vivo model of PD.

## 4. Materials and Methods

### 4.1. Materials and Equipment

The human neuroblastoma cell line SH-SY5Y (ATCC CRL-2266) was obtained from American Type Culture Collection (ATCC, Rockville, MD, USA). Dulbecco’s Modified Eagle Medium Nutrient Mixture F-12 (DMEM/F12) without Phenol Red (cat. no. 11039-021), Dulbecco’s Phosphate Buffered Saline 1× with Ca^2+^ and Mg^2+^ ions, cell culture 75 cm^2^ flasks (cat. no. 156499), transparent 96-well culture plates (cat. no. 655980) and black (cat. no. 655986) and white (cat. no. 655983) 96-well plates with optical bottoms were purchased from Greiner Bio-One (Kremsmünster, Austria). Fetal bovine serum (FBS, cat. no. 04-001-1A), 10× Trypsin-EDTA solution (cat. no. 03-051-5B), PBS without Ca^2+^ and Mg^2+^ ions (cat. no. 02-023-1A), and Penicillin-Streptomycin solution (cat. no. 03-031-1B) were obtained from Biological Industries (Cromwell, CT, USA).

Tetrahydrofurane (cat. no. 401757), 3,3-diethoxypropanol (cat. no. 273252), chloromethylstyrene (cat. no. 126136), ethylene oxide (cat. no. 743593), methanesulfonyl chloride (cat. no. 471259), triethylamine (cat. no. 471283), 2-propanol (cat. no. 278475), benzene (cat. no. 401765), chloroform (cat. no. 288306), hexane (cat. no. 296090), *n*-propylamine (cat. no. 240958), sodium sulfate (cat. no. 239313), 1,4-dithiothreitol (cat. no. 111474), 4-amino-2,2,6,6-tetramethylpiperidinyloxy (4-amino-TEMPO; cat. no. 163945) and potassium *O*-ethyldithiocarbonate (cat. no. 820744) were from Merck (Warsaw, Poland). 2,2′-Azo-*bis*-isobutyronitrile was obtained from Nouryon, (Amsterdam, Netherlands). Dialysis membranes, molecular cutoff 2 kD (cat. no. 888-11452) were from Spectrum, New Brunswick, NJ, USA.

The tetrazolium dye 3-(4,5-dimethylthiazol-2-yl)-2,5-diphenyltetrazolium bromide (MTT, cat. no. M2128), Trypan Blue solution (0.4%; cat. no. T8154), 6-hydroxydopamine hydrobromide (6-OHDA) (cat. no. 162957), L-ascorbic acid (cat. no. A0278), acridine orange 10-nonyl bromide (NAO, cat. no. A7847), trichloroacetic acid (TCA; cat. no. T4885), diethylenetriaminepentaacetic acid (DTPA; cat. no. D6518) and *o*-phthaldialdehyde (OPA; P0657) were provided by Sigma Aldrich (St. Louis, MO, USA). CellTiter-Glo^®^ Luminescent Cell Viability Assay (cat. no. G7571) and RealTime-Glo™ Annexin V Apoptosis and Necrosis Assay (cat. no. JA1011) were purchased from Promega (Madison, WI, USA). JC-1 Mitochondrial Membrane Potential Assay Kit was obtained from Abnova (Taiwan, China). PrestoBlue™ Cell Viability Reagent was purchased from ThermoFisher Scientific (Waltham, MA USA).

6-Hydroxydopamine hydrobromide (6-OHDA) (Sigma-Aldrich, cat. no. 162957) was freshly prepared and stabilized with 0.01% L-ascorbic acid, and filtered using 0.22 μm syringe filter for each experiment.

Water was purified with a Milli-Q system (Millipore, Bedford, MA, USA). Measurements of absorbance, fluorescence, and luminescence were carried out with Tecan Spark multimode microplate reader (Tecan Group Ltd., Männedorf, Switzerland).

### 4.2. Synthesis of Redox Nanoparticles

Acetal-poly(ethylene glycol)-mercapto (acetal-PEG-SH) polymer containing acetyl and mercapto terminals was prepared according to the procedure described by Akiyama et al. [32] with small modifications. Briefly, 15 mL of dry degassed tetrahydrofurane, 148 mg of 3,3-diethoxypropanol, and 186 mg of potassium 3,3-diethoxypropanolate were introduced into a reactor purged with argon and degassed several times to form potassium 3,3-diethoxypropanolate. The mixture was stirred for 15 min and 5.24 mL of ethylene oxide cooled at −20 °C were introduced via a cooled syringe. The mixture was allowed to react at room temperature (21 °C) for two days. Then, 928 µL of methanesulfonyl chloride and 2.09 mL of triethylamine were added, and the mixture was stirred at room temperature for 6 h. The polymer formed was precipitated with 500 mL of 2-propanol cooled at −20 °C, sedimented by centrifugation (5000× *g*, 30 min), washed 3 times with cold 2-propanol and freeze-dried with benzene. In addition, 10 mL of dry tetrahydrofurane and 33.6 g of potassium *O*-ethyldithiocarbonate in dry tetrahydrofurane/dimethylformamide (10 mL:5 mL) were successively added to 4.2 g of the polymer and stirred at room temperature for 3 h. The reaction mixture was added with chloroform and washed several times with saturated aqueous solution of NaCl. The organic phase was dried with sodium sulfate, concentrated by evaporation and recovered by precipitation with 500 mL of cold (−20 °C) 2-propanol and centrifugation; this cycle of precipitation/centrifugation was repeated thrice. Afterwards, the product was freeze-dried with benzene. The yield of the polymer was 3.4 g.

To generate sulphonyl groups, 3.4 g of acetal-PEG ethyldithiocarbonate was added with 719 µL of *n*-propylamine in 15 mL THF. The mixture was stirred at room temperature for 14 h. The product was precipitated in 500 mL of cold (−20 °C) 2-propanol, sedimented by centrifugation (5000× *g*, 30 min), washed with cold 2-propanol and sedimented thrice, and freeze-dried with benzene. To reduce dimers of acetal-PEG-SS-PEG acetal, 3.3 g of the polymer was treated with 1.0 g of 1,4-dithiothreitol in 25 mL of tetrahydrofurane. The product was recovered in 500 mL of cold 2-propanol, sedimented by centrifugation (5000× *g*, 30 min), washed 3 times with cold 2-propanol, and freeze-dried with benzene, yielding 2.7 g of the product (acetal-PEG-SH).

Further synthesis was performed according to Yoshitomi et al. [17]. In addition, 700 mg of acetal-PEG-SH was weighed into a flask, which was then degassed and purged with argon three times. Then, 1.35 mL of chloromethylstyrene, 16 mg of 2,2′-azobisisobutyronitrile and 10 of mL benzene was added to the flask, and polymerization was conducted at 60 °C for 24 h in a water bath. The product was recovered by precipitation with 400 mL of hexane and freeze-dried with benzene, washed three times with diethyl ether to eliminate the PCMS homopolymer, and freeze-dried with benzene. Furthermore, 892 mg of so obtained acetal-PEG-b-PCMS was weighed into a flask, added with 45 mL of a dimethylsulfoxide solution of 4-amino-TEMPO (1.962 g) and allowed for reacting at room temperature with stirring for 5 h. The product was precipitated by addition of 220 mL of cold (−20 °C) 2-propanol and centrifuged (5000× *g*, 30 min). The precipitation–centrifugation cycle was repeated 3 times, and the product was freeze-dried with benzene. In addition, 923 mg of the product was obtained.

For preparation of the core-shell-type nanoparticles from the copolymer, 50 mg of the product was dissolved in 5 mL of dimethylformamide, and the polymer solution was transferred into a preswollen membrane tube (Spectra/Por; molecular-weight cutoff size: 2 kD) and then dialyzed for 24 h against 2 L of water, which was changed three times.

### 4.3. SH-SY5Y Cell Culture

SH-SY5Y cell line was cultured in DMEM/F12 without phenol red, supplemented with 10% *v*/*v* heat-inactivated fetal bovine serum (hi-FBS) and 1% *v*/*v* penicillin and streptomycin solution. Cells were maintained at 37 °C in 5% carbon dioxide and 95% humidity. The cellular morphology was examined under an inverted microscope with phase contrast Zeiss Primo Vert (Oberkochen, Germany), cell viability was estimated by Trypan Blue exclusion test, and cells were counted using a Thoma hemocytometer (Superior Marienfeld, Lauda-Königshofen, Germany).

### 4.4. Cell Viability Assay

Cell viability was assayed with PrestoBlue or MTT. Human neuroblastoma cells were seeded in 96-well clear plate at a density of 3.5 × 10^4^ cells/well in 100 μL culture medium. After incubation, medium was gently removed by suction and replaced with 100 μL of 1 × PrestoBlue™ Cell Viability Reagent in PBS. After 1-h incubation in a cell culture incubator in the dark, absorbance was read at 570 nm, using 600 nm as a reference wavelength. 

Human neuroblastoma cells were seeded in 96-well clear plate at a density of 3.5 × 10^4^ cells/well in 100 μL culture medium. After appropriate incubation time (usually 24 h), medium was gently removed by suction and replaced with cell culture medium brought to appropriate pH or supplemented with appropriate compounds, brought up to appropriate pH. After the exposure, the medium was removed and replaced with 100 μL of 1 × PrestoBlue™ Cell Viability Reagent or 0.5 mg/mL of MTT solution in 1 × PBS with calcium and magnesium ions. In the PrestoBlue method, the cells were incubated in a CO_2_ incubator in the dark for 1 h and absorbance was read at 570 nm, using 600 nm as a reference wavelength. In the MTT method, the cells were incubated for 4 h in a CO_2_ incubator. Next, 100 μL/well of 2-propanol: HCl (250:1 *v*/*v*) solution were added to the cells in order to dissolve formazan crystals and shaken thoroughly for about 20–30 min. Absorbance was measured at 570 nm.

### 4.5. Treatment of SH-SY5Y Cells with Nanoparticles

For the analysis of protective properties of the NRNPs ^pH^, cells were seeded as described above in the previous paragraph. After overnight incubation to allow cell adherence, the medium was replaced with 50 μL/well of NRNPs ^pH^ in the medium adjusted with 1 M HCl to appropriate pH. Subsequently to 2-h preincubation with antioxidant, 50 μL/well of 130 μM 6-OHDA were added (final concentration: 65 μM) and then incubated for 24 h. Cells cultured in complete medium served as a negative control, whereas cells treated with 6-OHDA were considered as a positive control.

### 4.6. Analysis of Penetration of pH-Sensitive NRNPs into SH-SY5Y Cells

Cells from highly confluent T-75 flask were trypsinized, centrifuged (5 min, 900 rpm), and resuspended in 3 mL of medium in four parts, three 250-µL samples for each part. Each part was centrifuged again and the medium was replaced with 250 µL of cell medium of different pH (7.4, 6.5, 6.0 and 5.5, respectively), containing 30 μM NRNPs ^pH^. Cells were incubated for 6 h. After incubation, the cells were centrifuged, and the supernatant was collected and frozen for further examination. The pellet was washed with 1 mL of PBS and then centrifuged once again. The pellet was suspended in 50 μL/each sample of PBS and frozen. EPR signal intensity of nitroxide was measured.

### 4.7. Electron Spin Resonance (ESR) Spectroscopy Measurements

ESR signal intensity of nitroxide residues within NRNPs ^pH^ (~15 μL) was measured using microhematocrit capillaries (nonheparinized microhematocrit tubes; 1.55 × 75 mm; Medlab Products, Raszyn, Poland) in a Bruker multifrequency and multiresonance FT-EPR ELEXSYS E580 apparatus (Bruker BioSpin, Billerica, MA, USA). The spectrometer was operated at X-band (around 9.4 GHz). The following settings were used: central field, around 3354.0 G; modulation amplitude, 0.3 G; modulation frequency, 100 kHz; microwave power, 94.64 mW; power attenuation 2.0 dB; scan range, 100 G; conversion time, 25 ms; and sweep time, 25.6 s. The spectra were recorded with 1024 points per scan. The spectra were recorded and analyzed using Xepr 2.6b.74 software. The signal was integrated twice to determine its area and thus the concentration of the radical.

### 4.8. Analysis of Time-Penetration Axis of pH-Sensitive NRNPs

In order to establish the optimal time of NPs internalization into cells, cells were prepared similarly as above, but cells were resuspended in medium of pH 6.5 and pH 7.4 containing NRNPs ^pH^ and incubated for 3 h, 6 h, 12 h, and 24 h.

### 4.9. Assessment of Intracellular ATP Level

The intracellular ATP level was estimated using CellTiter-Glo^®^ Luminescent Cell Viability Assay. Cells were seeded into white 96-well plate with optical bottom, cultured and treated as previously described (Section 4.4). Cells were tested after 24 h incubation with 6-OHDA by adding 100 μL of CellTiter-Glo^®^ Reagent to each well. The next steps were performed according to the manufacturer’s protocol.

### 4.10. Content of Reduced Glutathione

The content of reduced glutathione (GSH) was tested using *ortho*-phthalaldehyde (OPA) [33]. SH-SY5Y cells were seeded in a clear 96-well plate at amount of 4 × 10^4^ cells/well in 100 μL culture medium and treated adequately. GSH was measured after 24 h incubation with 6-OHDA. As follows, the medium was aspirated, and cells were washed with PBS (150 μL/well). Afterwards, wells were filled with 60 μL/well of a newly prepared cold lysis buffer (RQB buffer: the solution of 20 mM HCl, 5% TCA, 5 mM DTPA, 10 mM L-ascorbic acid); next, the plates were agitated at 900 rpm for 5 min and centrifuged at 4000 rpm (5 min).

The lysates were subsequently transferred into two black 96-well plates with black bottom in the amount of 25 μL/well, namely ‘+NEM’ and ‘−NEM’. Within the first plate ‘+NEM’, 4 μL/well of freshly prepared 7.5 mM NEM in cold RQB buffer were added. Then, 40 μL/well of 1 M phosphate buffer (pH 7.0) were added into both plates and shaken for 5 min at 900 rpm. Next, 160 μL/well of cold 0.1 M phosphate buffer (pH 6.8) and 25 μL/well of newly prepared 0.5% OPA in methanol were pipetted into both plates and shaken at 900 rpm for 30 min. Fluorescence was measured at 355/430 nm. GSH concentration was determined by subtracting the fluorescence of the (‘−NEM’) plate from the fluorescence of the (‘+NEM’) plate, and GSH content was calculated with respect to protein content in each well.

### 4.11. Protein Assay

Protein content was assayed according to Lowry et al. [34].

### 4.12. Estimation of ROS Levels Using DHE Fluorescent Probe

Cells were handled as described above. Cells were seeded onto black 96-well plates with a clear bottom. The test was performed after 24 h incubation with 6-OHDA. Then, 100 μL/well of freshly prepared DHE working solution in PBS were added; the final concentration of the probe was equaled to 10 μM. The fluorescence was measured immediately at 37 °C, at 475/579 nm for 2 h, at 1 min intervals.

### 4.13. Evaluation of Changes of Mitochondrial Membrane Potential (ΔΨ_m_)

Changes of mitochondrial membrane potential were evaluated using JC-1 (5,5,6,6-tetrachloro-1,1,3,3-tetraethylbenzimidazolylcarbocyanine iodide) with a Mitochondrial Membrane Potential Assay Kit. In mitochondria with high ΔΨ_m_, JC-1 forms complexes with profound red fluorescence, whereas, in mitochondria that exhibit low ΔΨ_m_ levels, JC-1 persists as monomers and exhibits exclusively green fluorescence.

The cells were seeded into black plates and treated as mentioned before. After 24 h incubation with drugs, the medium was discharged and replaced with 100× diluted JC-1 reagent in complete culture medium and incubated for 30 min in a CO_2_ incubator. Next, the plate was centrifuged at 4000 rpm for 5 min. Afterwards, the reagent was aspirated, and cells were washed with 150 μL/well Cell-Based Assay Buffer and centrifuged once more. After removing the supernatant, 100 μL/well of the new buffer was added. Fluorescence was measured at 540/570 nm (red fluorescence) and 485/535 nm (green fluorescence). The results were presented as a green to red fluorescence intensity ratio.

### 4.14. Mitochondrial Mass Assessment

Cells were seeded at the density of 2 × 10^5^ cells/well onto a 24-well plate and cultured as stated before. Following 24-h exposure to 6-OHDA, the cells were trypsinized, counted, and transferred to separate Eppendorf tubes, and then centrifuged for 6 min at 3000 rpm. Subsequently, the supernatant was discharged, and the cells were washed with 1 mL of PBS and centrifuged again. Afterwards, 1 mL of 10 μM NAO solution in PBS was added into the samples, and incubated for 10 min in a CO_2_ incubator at 37 °C. Next, the cells were centrifuged and the pellet was washed with PBS, and resuspended in 300 μL of PBS. Each sample was transferred into a 96-well black plate (100 μL/well; 3 repetitions). Fluorescence was measured at 435/535 nm. The results were determined in relation to the cell count.

### 4.15. Apoptosis and Necrosis Assay

To examine the type of cell death caused by the treatment, the levels of apoptosis and necrosis were assayed using RealTime-Glo™ Annexin V Apoptosis and Necrosis Assay. Cells were seeded into white 96-well plate with optical bottom, cultured, and treated adequately as stated above. After 24 h, 100 μL of the freshly prepared reagent was added to each well according to manufacturer’s protocol. The fluorescence and luminescence were measured immediately according to the protocol.

### 4.16. Statistical Analysis

Kruskal–Wallis test or Student *t*-test were performed to estimate the differences between positive control and NRNP ^Ph^-treated cells; Mann–Whitney U test or paired sample Student *t*-test were performed to compare effects at different pH. *p* ≤ 0.05 was considered statistically significant. Statistical analysis of the data was performed using a STATISTICA software package (version 13.3, StatSoft Inc. 2016, Tulsa, OK, USA, http://www.statsoft.com).

## 5. Conclusions

Our results demonstrate that NRNPs ^pH^ can protect SH-SY5Y cells from 6-OHDA induced damage in a cellular model of PD at pH 6.5, which may prevail in the brain in regions affected by PD. This finding may have potential importance for potential applications of NRNPs ^pH^ in preclinical and perhaps clinical studies.

## Data Availability

Data supporting the results of this study shall, upon appropriate request, be available from the corresponding author.

## Sample Availability

Samples of the compounds are not available from the authors.
